# Senescent glia—bridging neuronal mitochondrial dysfunction and lipid accumulation in aging

**DOI:** 10.1093/lifemeta/loae031

**Published:** 2024-07-24

**Authors:** Joel F Reyes, Mahima Devarajan, Dongming Cai, Douglas G Mashek

**Affiliations:** N. Bud Grossman Center for Memory Research and Care, Department of Neurology, The University of Minnesota, Minneapolis, MN 55455, USA; Department of Biochemistry, Molecular Biology and Biophysics, University of Minnesota, MN 55455, USA; Department of Biochemistry, Molecular Biology and Biophysics, University of Minnesota, MN 55455, USA; N. Bud Grossman Center for Memory Research and Care, Department of Neurology, The University of Minnesota, Minneapolis, MN 55455, USA; Geriatric Research Education & Clinical Center, The Minneapolis VA Health Care System, Minneapolis, MN 55417, USA; Department of Biochemistry, Molecular Biology and Biophysics, University of Minnesota, MN 55455, USA; Division of Diabetes, Endocrinology and Metabolism, Department of Medicine, University of Minnesota, MN 55455, USA; Institute on the Biology of Aging and Metabolism, University of Minnesota, MN 55455, USA

**Identifying cellular mechanisms that underlie senescence development *in vivo* has been challenging in the field of aging research. In a recent article published in *Nature*, Byrns *et al.* identified a population of naturally occurring senescent glial cells that emerge in response to aging-associated neuronal mitochondrial dysfunction. These senescent glial cells promote the accumulation of lipid droplets (LDs) in non-senescent glial cells and can be targeted to extend healthspan.**

Cellular senescence is a state of irreversible growth arrest that cells enter in response to various stressors, including DNA damage, oxidative stress, and oncogenic signals. Senescent cells play several roles: they prevent the proliferation of damaged cells, thereby acting as a tumor-suppressive mechanism, but their accumulation over time contributes to aging and various age-related diseases through the secretion of a range of pro-inflammatory cytokines, chemokines, and proteases known as the senescence-associated secretory phenotype (SASP). The composition of the SASP is highly variable and dynamic depending on the factor that induces the senescence and the cell type involved.

In the central nervous system, glial cells—comprising astrocytes, oligodendrocytes, and microglia—are essential for maintaining neuronal health and function. However, in aging, senescent glial cells accumulate and contribute to age-related pathologies [1]. Furthermore, mouse models of neurodegeneration have provided evidence that clearance of senescent glial cells alleviates tau-dependent neurodegeneration and decreases inflammation as well as β-amyloid plaque size [2], further supporting the role of senescence in neurodegenerative processes.

**Figure 1:**
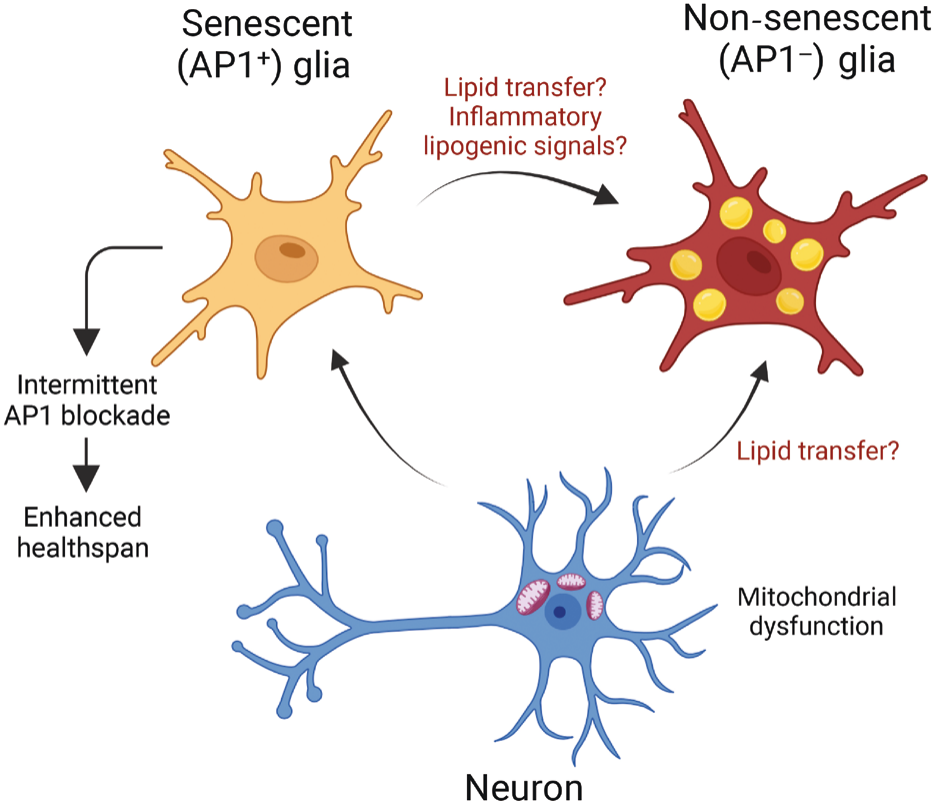
In response to aging-induced mitochondrial dysfunction, neurons promote activator protein 1 (AP1)-driven senescence in glia. Intermittent blockade of AP1 signaling in glia reduces lipid droplet (LD) accumulation in non-senescent glia and extends healthspan. AP1^+^ glia promote LD accumulation in non-senescent glia through speculative secretory mechanisms. The figure was made using BioRender.

While a rapidly growing body of literature supports the detrimental effects of senescence on aging-related declines in tissue function including neurodegeneration, the findings of the current study highlight the role of the transcription factor activator protein 1 (AP1) as a critical regulator of glial cell senescence during natural aging [3] (Fig. 1). AP1 has been characterized as a major regulator of the transcriptional program that drives senescence [4] and can drive tau pathology in glial cells [5]. However, mechanisms underpinning the formation of these senescence-like AP1^+^ glial cells remain elusive.

In their most recent work, Byrns *et al*. sought to characterize the functional roles of these AP1^+^ glial cells in aged *Drosophila* brain. Using a transgenic line expressing dsRed under the control of an AP1 binding motif to monitor the appearance and distribution of AP1^+^ glia, it was found that AP1^+^ glia accumulated in a regionally progressive manner as the brain ages. The senescence-associated features of AP1^+^ glia were confirmed by elevated β-galactosidase (SA-β-Gal) activity, increased DNA damage marker γH2Av (the phosphorylated histone variant H2Av), and increased expression of cell cycle arrest and pro-inflammatory genes. The comparison of gene expression profiles between young (5 days) and aged (40 days) neurons suggested an upregulated expression of inflammatory genes and downregulated genes in pathways related to mitochondrial function.

Mitochondrial dysfunction is a hallmark of aging, and its resulting oxidative stress can drive senescence [6]. Thus, to further investigate whether this mitochondrial dysfunction in neurons contributes to glial senescence, the authors performed a targeted RNAi screen against the inner mitochondrial complex genes in neurons using a drug-inducible (RU-486) neuron-specific GAL4 line with a fluorescent AP1 reporter. Knockdown of several mitochondrial complex genes led to increased dsRed expression in glia, mirroring patterns seen in aged flies, and further supporting a role for neuronal mitochondrial dysfunction in driving glial AP1 activity and senescence. Moreover, studies suggest that intermittent glial AP1 blockade decreased AP1-target genes and senescence biomarkers without any changes in markers of mitochondrial status, suggesting glial senescence as a downstream response to neuronal mitochondrial dysfunction.

Byrns *et al*. further explored the effects of blocking glial AP1 activity on the lifespan and healthspan of *Drosophila*. Consistent with studies employing genetic (mouse) and small molecule (mouse and human) ablation of senescent cells, intermittent targeting of AP1 senescent glial cells in *Drosophila* improved lifespan and healthspan, highlighting a significant role of glial senescence in regulating aging (Fig. 1). The results that periodic dampening of AP1 activity in glial cells can yield benefits implicate a potential avenue for interventions aimed at promoting healthy aging and longevity in other organisms.

This study corroborates and adds to the growing body of data showing that senescence is, at least in part, a metabolically driven process. Byrns *et al*. showed that neuronal mitochondrial dysfunction is the initiating factor in the development of glial cell senescence and lipid accumulation, consistent with other work in flies and mammals [7]. This implicates neuronal mitochondrial function as a regulator of lipid transport from neurons to glia, which has been shown to occur via apolipoprotein E (ApoE)-mediated transport of fatty acids and is thought to protect neurons from oxidative stress [8].

Interestingly, while lipid droplet (LD) accumulation is associated with oxidative stress and it has been shown that LDs accumulate in senescent cells and inflammatory glia [9], Byrns *et al*. found low LD content in AP1^+^ senescent cells (which are characterized by oxidative stress and inflammation). In fact, it is the AP1^−^ non-senescent glia that accumulated LDs in response to neuronal mitochondrial dysfunction. The authors presented an intriguing nuance of the consequences of this LD accumulation—while LD accumulation can be detrimental, they showed that ablation of LDs through suppressing lipogenesis or increasing lipolysis reduced lifespan and increased oxidative stress, indicating their protective value. They then showed that intermittent ablation of AP1^+^ senescent cells reduced LD abundance, and while these brains showed increased oxidative stress, the flies still showed enhanced healthspan and lifespan. Consistent with these findings, triglyceride lipase overexpression in *Drosophila* enhances numerous markers of healthspan, despite showing susceptibility to oxidative stress [10]. Taken together, this indicates that LD accumulation, much like senescent cell accumulation, is a cellular response that is either detrimental or beneficial in a context-dependent manner.

While studies often focus on the presence or absence of LDs as an indicator of disease or cellular dysfunction, alterations in both the protein and lipid compositions of LDs and the rates of lipid flux through the LD storage pool are likely better indicators of cellular function, as these regulate the signaling and capacity to store lipids to prevent lipotoxicity during times of high-lipid exposure. The Byrns *et al*. study revealed that neurons and AP1^+^ and AP1^−^ glia had robust differences in lipid composition; compared with AP1^−^ microglia, AP1^+^ microglia had an increase in free fatty acids, cholesterol esters, ceramides, and phosphatidylethanolamine, whereas AP1^−^ cells were enriched in triacylglycerol (consistent with more LDs) and several phospholipid species. While changes in lipid composition undoubtedly impact cellular function, changes in LD proteins or lipid transport/signaling likely contribute to the discordance between LD abundance and inflammation in the glial cell subpopulations and in response to manipulations of AP1 or lipid metabolism. For instance, Byrns *et al*. showed that a secreted factor(s) from senescent cells can promote LD accumulation in non-senescent cells in an AP1/Jun2-dependent manner. Inflammatory signals are well known to induce LD accumulation (in many cases, do so in an LD-protein-dependent manner) in numerous cell types and could underlie these observed effects. Alternatively, AP1^+^ glia may be secreting lipids that are taken up and accumulate in neighboring AP1^−^ glia, much like the mechanism of neuronal lipid secretion to protect neurons from oxidative stress. Future studies defining this cellular crosstalk are needed to help us understand the cellular heterogeneity of neurodegenerative diseases and identify precise glial cell populations that can be therapeutically targeted. More importantly, confirmatory studies in animal models and human brain samples about the functional relevance of AP1 and its dependent factors in senescence and the aging process are needed. Regardless, Bryns *et al*. reinforce the growing body of literature showing that senescence and alterations in metabolism are key factors contributing to aging-related neurodegeneration.
